# Supplementary material for: Deep Mixture of Linear Mixed Models for Complex Longitudinal Data
Source: Stat Med. 2025 Oct 7;44(23-24):e70288. doi: 10.1002/sim.70288 (PMC12503021; doi:10.1002/sim.70288)
Supplement: Supplementary file 1 — Data S1: Additional supporting information including the formal mathematical description of the DMLMM (A), details on the variational Bayes approach for posterior inference (B), and an application to missing data imputation for gene expression data not discussed within the main text (C) may be found in the online version of the article at the publisher's website. Python code for the DMLMM is publicly available github.com/kocklucx/DMLMM. [file SIM-44-0-s001.pdf]

Supporting Information for
"Deep mixture of linear mixed models for complex longitudinal data"

by

Lucas Kock, Nadja Klein and David J. Nott

Content

A Formal description of the DMLMM

B Details on posterior computation

B.1 Mean field variational approximation for the DMLMM . . . .

B.2 Derivation of the ELBO . . . .

C Missing data imputation for gene expression data

A Formal description of the DMLMM

Let y_{ij} denote the jth observation for subject i taken at time t_{ij}, $i = 1, \ldots, n$, $j = 1, \ldots, n_i$. Denote the multivariate response vector of all observations for subject i as $y_i = (y_{i1}, \ldots, y_{in_i})^\top \in \mathbb{R}^{n_i}$ with corresponding vector of time points $t_i = (t_{i1}, \ldots, t_{in_i})$. Writing $\beta_i = z_i^{(0)}$, the full DMLMM can be written as

$$y_i = B(t_i) z_i^{(0)} + \varepsilon_i, \tag{1}$$

where latent variables $z_i^{(l)}$, at layer $l = 1, \ldots, L$ are generated according to the following generative process. Let $K^{(l)}$ denote the number of mixture components at layer l. Then, with probability $w_k^{(l)}$, $k = 1, \ldots, K^{(l)}$, $\sum_k w_k^{(l)} = 1$, $z_i^{(l-1)}$ is generated as

$$z_i^{(l-1)} = \mu_k^{(l)} + B_k^{(l)} z_i^{(l)} + \epsilon_{ik}^{(l)}, \tag{2}$$

where $\epsilon_{ik}^{(l)} \sim \mathcal{N}(0, \delta_k^{(l)})$, $\mu_k^{(l)}$ is a $D^{(l-1)}$-vector, $B_k^{(l)}$ is a $D^{(l-1)} \times D^{(l)}$ factor loading matrix, $\delta_k^{(l)} = \text{diag}(\delta_{k1}^{(l)}, \ldots, \delta_{kD^{(l-1)}}^{(l)})$ is a $D^{(l-1)} \times D^{(l-1)}$ diagonal matrix with diagonal elements $\delta_{kj}^{(l)} > 0$ and $z_i^{(L)} \sim \mathcal{N}(0, I_{D^{(L)}})$. $\varepsilon_i \sim \mathcal{N}(0, \sigma^2 I_{n_i})$ is a vector of independent error terms.

As in Kock et al. (2022) we write

$$\begin{aligned}
z^{(l)} &= ({z_1^{(l)}}^\top, \ldots, {z_n^{(l)}}^\top)^\top, & z &= ({z^{(0)}}^\top, \ldots, {z^{(L)}}^\top)^\top, \\
\mu^{(l)} &= ({\mu_1^{(l)}}^\top, \ldots, {\mu_{K^{(l)}}^{(l)}}^\top)^\top, & \mu &= ({\mu^{(1)}}^\top, \ldots, {\mu^{(L)}}^\top)^\top, \\
B^{(l)} &= (\text{vec}(B_1^{(l)})^\top, \ldots, \text{vec}(B_{K^{(l)}}^{(l)})^\top)^\top, & B &= ({B^{(1)}}^\top, \ldots, {B^{(L)}}^\top)^\top, \\
\delta^{(l)} &= ({\delta_1^{(l)}}^\top, \ldots, {\delta_{K^{(l)}}^{(l)}}^\top)^\top & \delta &= ({\delta^{(1)}}^\top, \ldots, {\delta^{(L)}}^\top)^\top, \\
w^{(l)} &= (w_1^{(l)}, \ldots, w_{K^{(l)}}^{(l)})^\top, & w &= ({w^{(1)}}^\top, \ldots, {w^{(L)}}^\top)^\top,
\end{aligned}$$

where $\text{vec}(B_k^{(l)})$ denotes the vectorization of $B_k^{(l)}$. Also, we denote $\gamma_{ik}^{(l)} = 1$ if $z_i^{(l-1)}$ is generated from the k-th component model with probability $w_k^{(l)}$, $\gamma_{ik}^{(l)} = 0$ otherwise, and

write

$$\gamma = (\gamma^{(1)^\top}, \dots, \gamma^{(L)^\top})^\top, \quad \gamma^{(l)} = (\gamma_1^{(l)^\top}, \dots, \gamma_n^{(l)^\top})^\top, \quad \gamma_i^{(l)} = (\gamma_{i1}^{(l)}, \dots, \gamma_{iK^{(l)}}^{(l)})^\top.$$

We express all priors hierarchical and the full prior structure is given as

$$\begin{aligned}
\mathrm{vec}(B_k^{(l)})_j|\tau_k^{(l)}, h_{kj}^{(l)} &\sim \mathcal{N}\left(0, \tau_k^{(l)}/h_{kj}^{(l)}\right), & w^{(l)} &\sim Dir(\rho_1^{(l)}, \dots, \rho_{K^{(l)}}^{(l)})^\top \\
\tau_k^{(l)}|\xi_k^{(l)} &\sim \mathcal{IG}\left(\frac{1}{2}, \frac{1}{\xi_k^{(l)}}\right), & \xi_k^{(l)} &\sim \mathcal{IG}\left(\frac{1}{2}, \frac{1}{(\nu^{(l)})^2}\right) \\
h_{kj}^{(l)}|c_{kj}^{(l)} &\sim \mathcal{G}\left(\frac{1}{2}, c_{kj}^{(l)}\right), & c_{kj}^{(l)} &\sim \mathcal{G}\left(\frac{1}{2}, 1\right) \\
\mu_{kj}^{(l)}|g_{kj}^{(l)} &\sim \mathcal{N}(0, G^{(l)} g_{kj}^{(l)}), & g_{kj}^{(l)} &\sim \mathcal{IG}\left(\frac{1}{2}, \frac{1}{2}\right) \\
\delta_{kj}^{(l)}|\psi_{kj}^{(l)} &\sim \mathcal{IG}\left(\frac{1}{2}, \frac{1}{\psi_{kj}^{(l)}}\right), & \psi_{kj}^{(l)} &\sim \mathcal{IG}\left(\frac{1}{2}, \frac{1}{{A^{(l)}}^2}\right) \\
\sigma^2|\psi^{(0)} &\sim \mathcal{IG}\left(\frac{1}{2}, \frac{1}{\psi^{(0)}}\right), & \psi^{(0)} &\sim \mathcal{IG}\left(\frac{1}{2}, \frac{1}{{A^{(0)}}^2}\right).
\end{aligned}$$

$\nu = (\nu^{(1)}, \dots, \nu^{(L)})^\top$, $A = (A^{(0)}, A^{(1)}, \dots, A^{(L)})^\top$ and $\rho = (\rho^{(1)^\top}, \dots, \rho^{(L)^\top})^\top$ with $\rho^{(l)} = (\rho_1^{(l)}, \dots, \rho_{K^{(l)}}^{(l)})^\top$ are known hyperparameters and we write

$$\begin{aligned}
g &= (g^{(1)^\top}, \dots, g^{(L)^\top})^\top, & g^{(l)} &= (g_1^{(l)^\top}, \dots, g_{K^{(l)}}^{(l)^\top})^\top, & g_k^{(l)} &= (g_{k1}^{(l)}, \dots, g_{kD^{(l-1)}}^{(l)})^\top \\
h &= (h^{(1)^\top}, \dots, h^{(L)^\top})^\top, & h^{(l)} &= (h_1^{(l)^\top}, \dots, h_{K^{(l)}}^{(l)^\top})^\top, & h_k^{(l)} &= (h_{k1}^{(l)}, \dots, h_{k\kappa^{(l)}}^{(l)})^\top, \\
c &= (c^{(1)^\top}, \dots, c^{(L)^\top})^\top, & c^{(l)} &= (c_1^{(l)^\top}, \dots, c_{K^{(l)}}^{(l)^\top})^\top, & c_k^{(l)} &= (c_{k1}^{(l)}, \dots, c_{k\kappa^{(l)}}^{(l)})^\top, \\
\psi &= (\psi^{(0)^\top}, \dots, \psi^{(L)^\top})^\top, & \psi^{(l)} &= (\psi_1^{(l)^\top}, \dots, \psi_{K^{(l)}}^{(l)^\top})^\top, & \psi_k^{(l)} &= (\psi_{k1}^{(l)}, \dots, \psi_{kD^{(l-1)}}^{(l)})^\top,
\end{aligned}$$

$$\begin{aligned}
\tau &= (\tau^{(1)^\top}, \dots, \tau^{(L)^\top})^\top, & \tau^{(l)} &= (\tau_1^{(l)^\top}, \dots, \tau_{K^{(l)}}^{(l)^\top})^\top, \\
\xi &= (\xi^{(1)^\top}, \dots, \xi^{(L)^\top})^\top, & \xi^{(l)} &= (\xi_1^{(l)^\top}, \dots, \xi_{K^{(l)}}^{(l)^\top})^\top,
\end{aligned}$$

where $\kappa^{(l)} = D^{(l-1)} D^{(l)}$.

B Details on posterior computation

In what follows we use the same notation as Kock et al. (2022).

B.1 Mean field variational approximation for the DMLMM

We consider the factorized form

$$q_\lambda(\theta) = q(\sigma^2)q(\mu)q(\mathrm{vec}(B))q(z)q(w)q(\delta)q(\gamma)q(\psi)q(g)q(h)q(c)q(\tau)q(\xi). \tag{3}$$

and each factor in (3) will also be fully factorized.

$$q(\sigma^2) = p_{\mathcal{IG}}(\sigma^2; a^{(0)}(\sigma^2), b^{(0)}(\sigma^2))$$

$$q(\mu) = \prod_{l=1}^{L}\prod_{k=1}^{K^{(l)}}\prod_{j=1}^{D^{(l-1)}} q(\mu_{kj}^{(l)}), \quad q(\mu_{kj}^{(l)}) = \phi(\mu_{kj}^{(l)}; m_{kj}^{(l)}(\mu), \sigma_{kj}^{(l)}(\mu)^2),$$

$$q(B) = \prod_{l=1}^{L}\prod_{k=1}^{K^{(l)}}\prod_{j=1}^{\kappa^{(l)}} q(\mathrm{vec}(B_k^{(l)})_j), \quad q(\mathrm{vec}(B_k^{(l)})_j) = \phi(\mathrm{vec}(B_k^{(l)})_j; m_{kj}^{(l)}(B), \sigma_{kj}^{(l)}(B)^2),$$

$$q(z) = \prod_{i=1}^{n}\prod_{l=0}^{L}\prod_{j=1}^{D^{(l)}} q(z_{ij}^{(l)}), \quad q(z_{ij}^{(l)}) = \phi(z_{ij}^{(l)}; m_{ij}^{(l)}(z), \sigma_{ij}^{(l)}(z)^2),$$

$$q(w) = \prod_{l=1}^{L} q(w^{(l)}) \quad q(w^{(l)}) = p_{Dir}(w^{(l)}; d_1^{(l)}, \ldots, d_{K^{(l)}}^{(l)}),$$

$$q(\delta) = \prod_{l=1}^{L}\prod_{k=1}^{K^{(l)}}\prod_{j=1}^{D^{(l-1)}} q(\delta_{kj}^{(l)}), \quad q(\delta_{kj}^{(l)}) = p_{\mathcal{IG}}(\delta_{kj}^{(l)}; a_{kj}^{(l)}(\delta), b_{kj}^{(l)}(\delta)),$$

$$q(\gamma) = \prod_{i=1}^{n}\prod_{l=1}^{L} q(\gamma_i^{(l)}), \quad q(\gamma_i^{(l)}) = p_{\mathcal{M}}(\gamma_i^{(l)}; 1, \alpha_{i1}^{(l)}, \ldots, \alpha_{iK^{(l)}}^{(l)})$$

$$q(\psi) = \prod_{l=0}^{L}\prod_{k=1}^{K^{(l)}}\prod_{j=1}^{D^{(l-1)}} q(\psi_{kj}^{(l)}), \quad q(\psi_{kj}^{(l)}) = p_{\mathcal{IG}}(\psi_{kj}^{(l)}; a_{kj}^{(l)}(\psi), b_{kj}^{(l)}(\psi))$$

$$q(g) = \prod_{l=1}^{L}\prod_{k=1}^{K^{(l)}}\prod_{j=1}^{D^{(l-1)}} q(g_{kj}^{(l)}), \quad q(g_{kj}^{(l)}) = p_{\mathcal{IG}}(g_{kj}^{(l)}; a_{kj}^{(l)}(g), b_{kj}^{(l)}(g)),$$

$$q(h) = \prod_{l=1}^{L}\prod_{k=1}^{K^{(l)}}\prod_{j=1}^{\kappa^{(l)}} q(h_{kj}^{(l)}), \quad q(h_{kj}^{(l)}) = p_{\mathcal{G}}(h_{kj}^{(l)}; a_{kj}^{(l)}(h), b_{kj}^{(l)}(h)),$$

$$q(c) = \prod_{l=1}^{L}\prod_{k=1}^{K^{(l)}}\prod_{j=1}^{\kappa^{(l)}} q(c_{kj}^{(l)}), \quad q(c_{kj}^{(l)}) = p_{\mathcal{G}}(c_{kj}^{(l)}; a_{kj}^{(l)}(c), b_{kj}^{(l)}(c)),$$

$$q(\tau) = \prod_{l=1}^{L} \prod_{k=1}^{K^{(l)}} q(\tau_k^{(l)}), \quad q(\tau_k^{(l)}) = p_{\mathcal{IG}}(\tau_k^{(l)}; a_k^{(l)}(\tau), b_k^{(l)}(\tau)),$$
$$q(\xi) = \prod_{l=1}^{L} \prod_{k=1}^{K^{(l)}} q(\xi_k^{(l)}), \quad q(\xi_k^{(l)}) = p_{\mathcal{IG}}(\xi_k^{(l)}; a_k^{(l)}(\xi), b_k^{(l)}(\xi)),$$

where $\phi(\cdot; \mu, \sigma^2)$ denotes the density of a univariate Gaussian distribution, $p_{\mathcal{IG}}(\cdot; a, b)/p_{\mathcal{G}}(\cdot; a, b)$ the densities of inverse gamma/gamma distributions with shape and scale a, b, respectively, $p_{Dir}(\cdot; d_1, \dots, d_K)$ is the density of a K-dimensional Dirichlet distribution and $p_{\mathcal{M}}(\cdot; 1, \alpha_1, \dots, \alpha_k)$ denotes the density of the multinomial distribution with one trial and probabilities $\alpha_1, \dots, \alpha_k$.

B.2 Derivation of the ELBO

The evidence lower bound is

$$\mathcal{L}(\lambda) = E_q(\log(p(\theta))) + E_q(\log(p(y|\theta))) - E_q(\log(q_\lambda(\theta))).$$

The ELBO can be can be decomposed as

$$\mathcal{L}(\lambda) = \mathcal{L}^{\text{DMFA}}(\lambda) + \mathcal{L}^{\text{Reg}}(\lambda),$$

where

$$\mathcal{L}^{\text{DMFA}}(\lambda) = \mathbb{E}q_\lambda \left[\sum_{i=1}^{n} \log(p(z_i^{(0)} \mid \theta_{\text{DMFA}})) + \log(p(\theta_{\text{DMFA}})) - \log(q_{\lambda_{\text{DMFA}}}(\theta_{\text{DMFA}})) \right]$$

and

$$\mathcal{L}^{\text{Reg}}(\lambda) = \mathbb{E}q_\lambda \left[\sum_{i=1}^{n} \log(p(y_i \mid z_i^{(0)}, \sigma^2)) + \log(p(\sigma^2 \mid \psi^{(0)}) p(\psi^{(0)})) - \log(q_{\lambda_{\text{Reg}}}(\theta_{\text{Reg}})) \right].$$

Then,

$$\begin{aligned}
&\mathbb{E}q_\lambda \left[\log(p(y_i \mid z_i^{(0)}, \sigma^2)) \right] \\
&= \mathbb{E}q_\lambda \left[-\frac{n_i}{2} \left(\log(2\pi) - \log(\sigma^2) \right) - \frac{1}{2\sigma^2} \left(y_i - B(t_i) z_i^{(0)} \right)^\top \left(y_i - B(t_i) z_i^{(0)} \right) \right] \\
&= -\frac{n_i}{2} \left(\log(2\pi) - \log(b^{(0)}(\sigma^2)) + \psi(a^{(0)}(\sigma^2)) \right) \\
&- \frac{a^{(0)}(\sigma^2)}{2b^{(0)}(\sigma^2)} \left(\sum_{j=1}^{d} \left((y_{ij} - (B(t_i))_j m_i^{(0)}(z))^2 + \sum_{r=1}^{D^{(1)}} (B(t_i))_{jr}^2 \sigma_{ir}^{(0)}(z)^2 \right) \right),
\end{aligned}$$

$$\mathbb{E}q_\lambda\left[\log p(\sigma^2|\psi^{(0)}))\right] = -\frac{1}{2}\left\{\log b^{(0)}(\psi) - \psi(a^{(0)}(\psi))\right\} - \log\Gamma\left(\frac{1}{2}\right) - \frac{3}{2}\left\{\log b^{(0)}(\sigma^2) - \psi(a^{(0)}(\sigma^2))\right\},$$

$$E_q\left[\log p(\psi^{(0)})\right] = -\frac{1}{2}\log(A^{(0)^2}) - \log\Gamma\left(\frac{1}{2}\right) - \frac{3}{2}\left\{\log b^{(0)}(\psi) - \psi(a^{(0)}(\psi)\right\} - \frac{a^{(0)}(\psi)}{b^{(0)}(\psi)}\frac{1}{A^{(0)^2}}.$$

Finally,

$$\begin{aligned}\mathbb{E}q_\lambda\left[-\log(q_{\lambda_{\text{Reg}}}(\theta_{\text{Reg}}))\right] =& \sum_{r=1}^{d}\log(2\pi e\sigma_{ir}^{(0)}(z)^2) \\ &- \left[(1 + a^{(0)}(\sigma^2)\psi(a^{(0)}(\sigma^2)) - a^{(0)}(\sigma^2) - \log\left\{b^{(0)}(\sigma^2)\Gamma(a^{(0)}(\sigma^2))\right\}\right] \\ &- \left[(1 + a^{(0)}(\psi))\psi(a^{(0)}(\psi)) - a^{(0)}(\psi) - \log\left\{b^{(0)}(\psi)\Gamma(a^{(0)}(\psi))\right\}\right].\end{aligned}$$

This gives an analytical form for $\mathcal{L}^{\text{Reg}}(\lambda)$. Since the DMLMM can be regarded as a DMFA model where the top layer is replaced by a regression layer, $\mathcal{L}^{\text{DMFA}}(\lambda)$ follows the expression of the ELBO for the DMFA model excluding the terms for the top layer. Calculations for all terms involved in $\mathcal{L}^{\text{DMFA}}(\lambda)$ are given in Web Appendix A.2 of Kock et al. (2022). Putting everything together, this results in an analytical expression for $\mathcal{L}(\lambda)$.

As for the DMFA model parameters split naturally into global $\lambda_G = (m(\mu), \sigma(\mu), m(B), \sigma(B), d, a(\sigma^2), b(\sigma^2), a(\delta), b(\delta), a(\psi), b(\psi), a(g), b(g), a(h), b(h), a(c), b(c), a(\tau), b(\tau), a(\xi), b(\xi))$ and local parameters $\lambda_L = (m(z), \sigma(z), \alpha)$ allowing to use the algorithm described in Web Appendix B of Kock et al. (2022).

C Missing data imputation for gene expression data

Missing data imputation in time-course gene expression data is an important task in modern genomics. Here, we consider a publicly available dataset describing the temporal expression of 1,444 genes in the 150-min cell cycle of the bacterium Caulobacter crescentus. RNA was harvested from cell samples at regular 15-min time intervals leading to a total of $15,884$ observations in the microarray denoted y_{ij}, where i indexes genes and j indexes observation times. A full description of the dataset is available in Laub et al. (2000).

Experimental design The original data frame does not contain any missing values, so we remove measurement of the ith gene at time t_j, y_{ij}, with probability α and call α the (induced) level of sparsity. Additionally, we enforce that for each gene y_i at least one measurement is available $n_i \geqslant 1$. This allows us to compare imputed values with the ground truth. An example of a sparsified dataset is given in Figure 1a). We repeat the experiment $M = 10$ times for each level of sparsity $\alpha = 0.2, \ldots, 0.8$. For the DMLMM the design matrices contain a B-spline basis with inner knots set at the 11 measurement times. We benchmark the DMLMM against popular imputation approaches including replacement with the row / column wise mean, the mean value from 10-th nearest neighbors (KNN) and imputation based on the singular value decomposition (SVD).

Results Figure 1 shows one example run with sparsity level $\alpha = 0.5$. We find that the DMLMM reasonably combines information of similarly behaving genes to impute missing values. Figure 2a) shows that point-wise credible intervals derived by DMLMM are well calibrated even for high levels of sparsity. In terms of RMSE, DMLMM is compatible with the benchmarks and performs best for levels of sparsity up to $\alpha = 0.6$ (Figure 2b)).

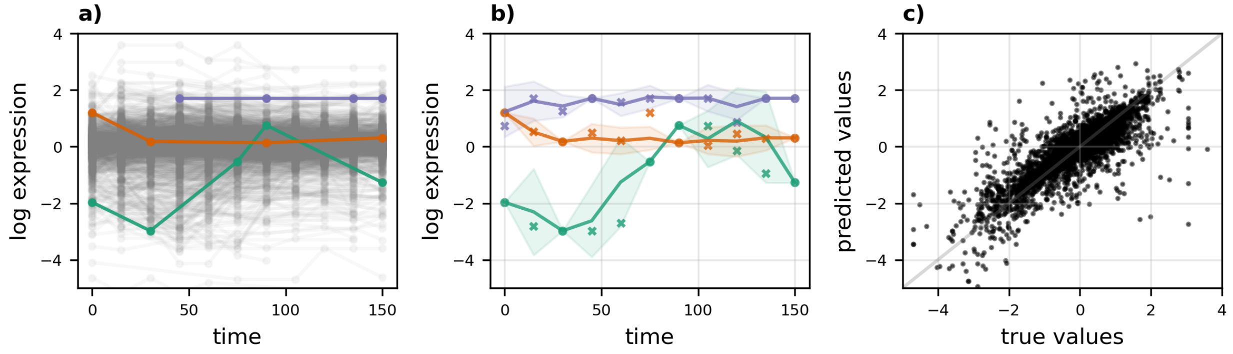

Figure 1: Gene expression data (50% sparse). Results from one randomly selected run. **a)** Spaghetti plot for all observed genes. Three randomly selected genes y_i are marked in color. **b)** Predicted trajectories (bold) and 95% credible intervals for the three randomly selected genes, with observed measurements given by dots and missing data given by crosses. **c)** Scatter plot of true missing values versus imputed values $\mathbb{E}(\tilde{y}_j \mid y_i, \hat{\eta})$.

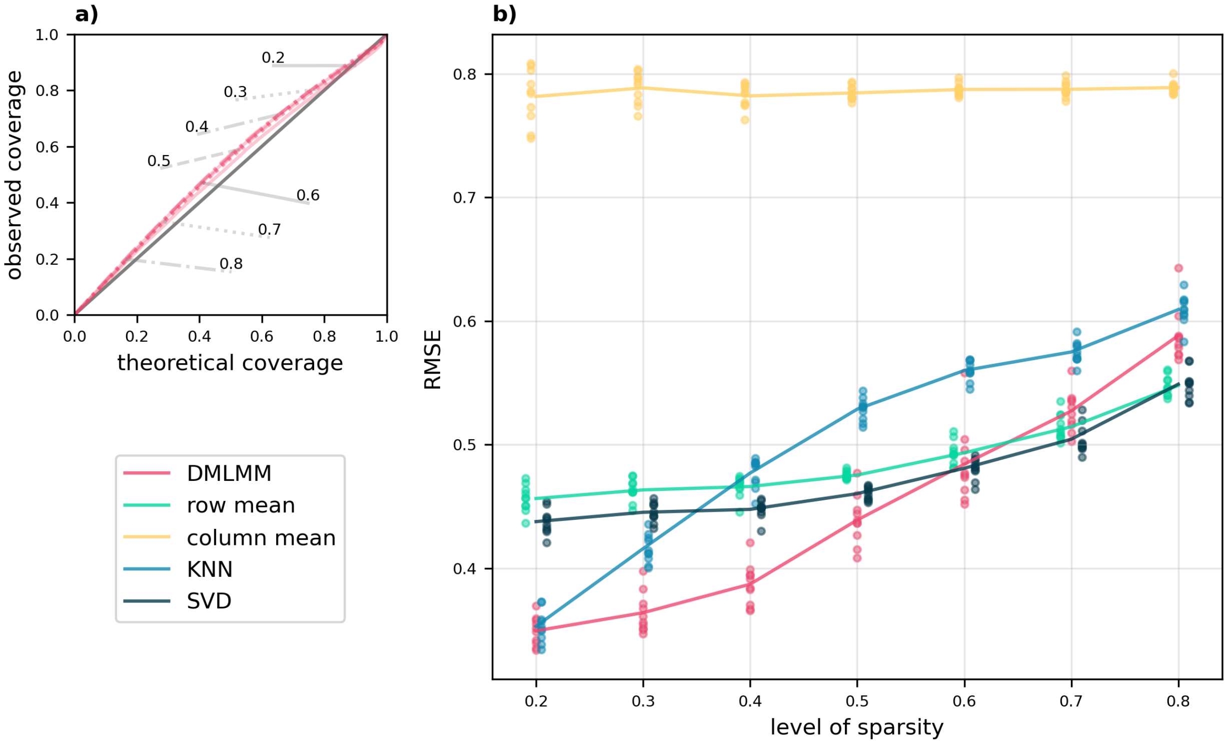

Figure 2: Gene expression data **a)** Observed coverage rates for point-wise symmetrical credible intervals for different levels of sparsity derived by DMLMM. **b)** RMSE (y-axis) for different levels of sparsity (x-axis). Individual runs are shown by dots, while the bold lines indicate the mean across all independent repetitions for DMLMM (red), row mean (green), column mean (yellow), KNN (blue) and SVD (grey).

References

Kock, L., Klein, N. and Nott, D. J. (2022). Variational inference and sparsity in high-dimensional deep Gaussian mixture models, *Statistics and Computing* **32**(5): 70.

Laub, M. T., McAdams, H. H., Feldblyum, T., Fraser, C. M. and Shapiro, L. (2000). Global analysis of the genetic network controlling a bacterial cell cycle, *Science* **290**(5499): 2144–2148.
